# A Comprehensive Review on Alcohol Abuse Disorder Fatality, from Alcohol Binges to Alcoholic Cardiomyopathy

**DOI:** 10.3390/diagnostics14111189

**Published:** 2024-06-05

**Authors:** Antonina Argo, Walter Pitingaro, Maria Puntarello, Roberto Buscemi, Ginevra Malta, Tommaso D’Anna, Giuseppe Davide Albano, Stefania Zerbo

**Affiliations:** Department of Health Promotion Sciences, Maternal and Child Care, Internal Medicine and Medical Specialties “Giuseppe D’Alessandro”, University of Palermo, Via del Vespro 133, 90127 Palermo, Italy; walter.pitingaro@community.unipa.it (W.P.); maria.puntarello@community.unipa.it (M.P.); medtox@unipa.it (R.B.); tommaso.danna@policlinico.pa.it (T.D.); giuseppedavide.albano@unipa.it (G.D.A.); stefania.zerbo@unipa.it (S.Z.)

**Keywords:** alcohol intake, cardiomyopathy, sudden death, arrhythmias, stroke, hypertension, gender, Takotsubo, forensic

## Abstract

Frequent and excessive consumption of alcohol, be it episodic or sustained misuse, ranks among the top causes of mortality globally. This comprehensive analysis seeks to elucidate how alcohol misuse precipitates death, with a particular focus on associated cardiac anomalies. Notably, the phenomenon of “Holiday Heart Syndrome”, linked to binge drinking, is recognized for inducing potentially fatal cardiac arrhythmias. Moreover, persistent alcohol consumption is implicated in the development of alcoholic cardiomyopathy, a condition that underlies heart failure and arrhythmic disturbances of the heart. Additionally, individuals undergoing withdrawal from alcohol frequently exhibit disruptions in normal heart rhythm, posing a risk of death. This review further delves into additional alcohol-related mortality factors, including the heightened likelihood of hypertension, cerebrovascular accidents (strokes), and the connection between excessive alcohol use and Takotsubo syndrome.

## 1. Introduction

Alcohol is a significant contributor to worldwide disease and a leading cause of preventable death, with 3 million deaths per year attributable to alcohol. Both the occasional consumption of alcohol and its chronic intake are associated with an increased risk of road accidents, workplace productivity losses, increased medical and mental health costs, and more significant rates of crime and violence [1]. Global alcohol abuse and high rates of consumption cause multiple health problems; alcohol is considered responsible for 5.3% of all deaths worldwide [2].

One of the characteristics that makes alcohol harmful is its systemic toxic effect, which determines macro- and microscopic alterations to the organs of the human body, as well as of the physiological systems of which they are part. Alcohol exerts its toxic effects on almost all organs of the human body, such as the gastrointestinal system, the central and peripheral nervous system, the cardiovascular system, and especially the liver, the organ responsible for metabolizing ethanol [3,4,5].

The potentially lethal effects of alcohol are related to the alteration in the normal heart rhythm induced by alcohol intake, evident both in occasional but excessive consumption, the so-called alcoholic binges, as well as in chronic consumption with the onset of alcoholic cardiomyopathy and complications related to it.

Despite the cited adverse health effects related to alcohol consumption, several observational studies have shown a significant reduction in all-cause cardiovascular mortality in subjects with low levels of alcohol consumption compared to abstainers [6,7,8]. A recent meta-analysis with more than 16,000 patients confirmed that daily alcohol consumption of up to 25 g/d was associated with a statistically significant reduction in the incidence of cardiovascular mortality [9].

Given the extensive scientific literature relating to the harmful effects of alcohol, both short and long-term, this comprehensive review aims to clarify and list the pathophysiology mechanisms responsible for alcohol-related deaths.

## 2. Materials and Methods

This literature review was conducted to identify articles regarding the potentially lethal effects of alcohol consumption on the heart.

### 2.1. Search Strategy

**Figure 1:**
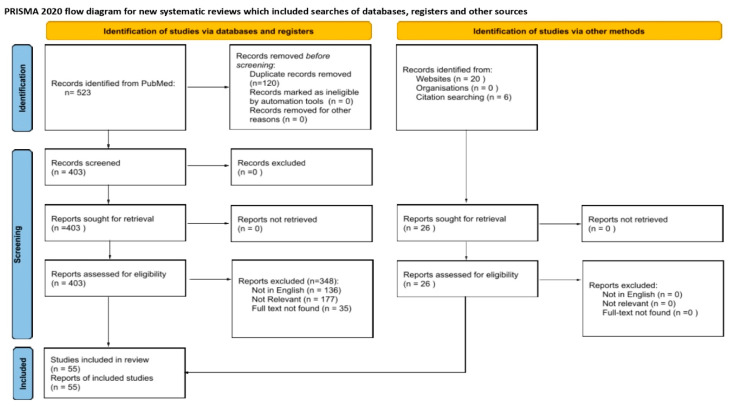
PRISMA flow diagram of literature review and study identification, screening, eligibility, inclusion and exclusion.

The literature review was conducted between October and November 2023, utilizing PubMed’s electronic database. The search employed Medical Subject Headings (MeSH), including “cardiomyopathy alcoholic”, “sudden death”, “cardiac arrhythmias”, “stroke”, “hypertension”, and “Takotsubo cardiomyopathy.” Boolean operators, specifically “AND” and “OR”, were applied to refine the search terms (refer to Figure 1). Additionally, the review extended to examining relevant websites and conducting citation searches, emphasizing subjects pertinent to our study [10].

### 2.2. Study Selection

In the initial phase of the search, publications were screened based on their titles and abstracts by three researchers, AA and WP and GM, according to predefined inclusion criteria: (1) articles published in English, (2) availability of full text, and (3) content relating to the cardiotoxic effects of alcohol. Exclusion criteria were applied to articles that were (1) not accessible in full, (2) repeated citations, or (3) unrelated to the scope of this review. All titles and abstracts meeting the inclusion criteria were retrieved in full text for further evaluation. These full-text articles were then independently reviewed by two investigators to determine their suitability for inclusion, with any discrepancies resolved through consensus. The search, spanning publications from 1965 to 2023, initially identified 523 studies. Of these, 120 were removed due to duplication, 177 for lack of relevance, 136 because they were not in English, and 35 due to the unavailability of the full text. Additionally, searches through websites and citations yielded 26 studies—20 identified through website searches and 6 through citation tracking. No studies from this supplementary search were excluded as all met the criteria of relevance, being in English, and having accessible full texts.

## 3. Results

The research team selected 55 studies for analysis, focusing on the connections between alcohol consumption and various cardiovascular conditions, including hypertension, stroke, cardiomyopathy, Takotsubo cardiomyopathy, cardiac arrhythmias, sudden death, and stroke: (1) (((((alcoholic cardiomyopathy) AND (cardiac arrhythmias)) AND (hypertension)) AND (stroke)) AND (sudden death)) AND (takotsubo cardiomyopathy); (2) (((((alcoholic cardiomyopathy) AND (cardiac arrhythmias)) AND (hypertension)) AND (stroke)) AND (sudden death)) AND (takotsubo cardiomyopathy); (3) (alcoholic cardiomyopathy) AND (cardiac arrhythmias); (4) (alcoholic cardiomyopathy) AND (hypertension); (5) (alcoholic cardiomyopathy) AND (stroke); (6) (alcoholic cardiomyopathy) AND (sudden death); (7) (alcoholic cardiomyopathy) AND (takotsubo cardiomyopathy). Additionally, the study incorporated findings from website and citation searches, through which an additional 26 studies were identified, 20 studies via website searches and 6 through citation searches. Each of these studies was evaluated for relevance, ensuring they were pertinent to the research topic, available in English, and accessible in full text. A comprehensive approach allowed us to review the latest scientific literature until November 2023, considering a total of 103 studies relating to the subject. The key characteristics and findings of the most relevant studies are summarized in Table 1, presenting a state-of-the-art overview of the impact of alcohol consumption on cardiovascular health from 39 studies.

## 4. Discussion

Alcoholic cardiomyopathy is a specific form of heart disease caused by long-term excessive alcohol consumption. Characterized by the weakening and thinning of the heart muscle, this condition impairs the heart’s ability to pump blood effectively, leading to a range of life-threatening complications. Over time, the heart struggles to maintain adequate blood flow to meet the body’s needs, resulting in symptoms such as shortness of breath, fatigue, swelling of the legs and feet (edema), and irregular heartbeats (arrhythmias). Alcoholic cardiomyopathy is a silent yet progressive disease; often, symptoms do not appear until the condition has advanced significantly. As the disease progresses, it can lead to heart failure, where the heart is unable to pump enough blood throughout the body, and other severe cardiovascular problems. Reducing alcohol intake or abstaining from alcohol altogether is critical in managing and potentially halting the progression of alcoholic cardiomyopathy.

A careful review of the scientific literature by three independent reviewers identified several pathophysiological conditions related to alcohol consumption. This could make an important contribution to the scientific community, not only for the medico-legal understanding of the causal mechanisms of alcohol-related deaths (in addition to the gross and microscopic findings and evidence from autopsy) but also in the view of a therapeutic perspective on alcohol-addicted patients.

### 4.1. Alcohol Consumption and Blood Pressure

The relationship between alcohol consumption and increased blood pressure was highlighted for the first time by Lian in 1915; he observed a greater prevalence of hypertension in subjects who had served in the military in the First World War and who consumed large quantities of alcohol [49]. The consumption of large quantities of alcohol is etiologically responsible for 16% of global cases of hypertension. The American Society of Hypertension has highlighted that consuming more than two drinks a day increases the risk of developing hypertension; furthermore, for each additional drink, an approximate increase in blood pressure of 1.5 mmHg was estimated [11]. Cross-sectional studies and meta-analyses have confirmed the increase in blood pressure resulting from alcohol consumption in a dose-dependent manner. The Cochrane Hypertension Information Specialist observed that alcohol causes transient vasodilation, which determines an initial reduction in blood pressure, probably mediated by atrial natriuretic peptide; this antihypertensive effect manifests itself within 6–12 h after alcohol intake, but subsequently, an increase in blood pressure values is observed [12,50]. Ethanol acts directly on smooth muscle cells and, by exerting its vasodilatory effects, causes hypotension. The two proposed mechanisms responsible for the vasodilatory effect of alcohol are alteration in the normal vasomotor tone of smooth muscle cells and reduction in the physiological contractile response of vascular smooth muscle cells to endogenous neurohormonal stimuli, which play a crucial role in maintaining vascular tone and peripheral regulation of blood flow [13,14]. Moreover, studies have elucidated that alcohol alters cytoplasmic calcium levels, thus directly affecting the normal contractile function of vascular smooth muscle cells. As previously discussed, alcohol plays a significant role in the development of arterial hypertension; notably, between 30 to 60% of individuals with a chronic alcoholism history suffer from hypertension [15]. Even at low plasma concentrations, alcohol enhances the hypertensive effects of substances such as vasopressin, catecholamines, and angiotensin II. Additionally, a mechanism by which alcohol contributes to hypertension involves alcohol-induced hypomagnesemia, leading to calcium accumulation in various tissues, including vascular smooth muscle cells [13,51]. Elevation in circulating catecholamines, associated with hypomagnesemia, represents other factors favoring the onset of hypertension in subjects with a history of chronic alcoholism. Researchers have highlighted the relationship between the onset of hypertension from alcohol consumption and sex; in fact, the consumption of 1–2 drinks in a single episode causes an increase in systolic pressure of 4–7 mmHg and of 4–6 mmHg in diastolic pressure in men. In contrast, in women, the consumption of the same quantity of alcohol is not correlated with an appreciable increase in blood pressure [15]. In a meta-analysis of 18 research groups, Roerecke and colleagues highlighted that the consumption of 1–2 drinks per day in female subjects, with an average age of 46.7 years, was not connected to an increase in the risk of developing hypertension, compared to abstainers. These results on daily alcohol consumption show a different relationship depending on gender; there is a linear relationship for men and a J-shaped relationship for women. If for men the consumption of any quantity of alcohol is associated with an increased risk of hypertension, on the contrary, for women the consumption of 1–2 drinks a day does not influence blood pressure values [16]. A meta-analysis involving several publications (USA, Japan, Korea) found a linear relationship between hypertension and daily alcohol consumption; the researchers calculated a relative risk of 1.7 in subjects who consumed four drinks per day and 2.5 in subjects who consumed eight drinks per day [11]. Klatsky and colleagues studied blood pressure values in 83,947 subjects with alcoholic habits. They divided these subjects into three categories of types of daily alcohol consumption (<2 drinks, between 3 and 5, >6 drinks). The researchers highlighted that male subjects who consumed <2 drinks per day had blood pressure levels identical to controls, while women who consumed the same amount of alcohol had lower blood pressure values than controls [17]. Finally, it is worth underlining how alcohol consumption in subjects taking anti-hypertensive drug therapy can negatively modify the pharmacological effect of the treatment [18,19]. Although this topic will be discussed later, it is important to highlight how both alcohol consumption and the resulting hypertension are etiological factors in the onset of alcoholic cardiomyopathy [52].

Alcoholic cardiomyopathy is defined as a pathological condition with structural, anatomical, and functional changes of the heart that develops in subjects who have consumed approximately 80–90 g of alcohol per day for at least five years [3,49].

The international scientific community has researched whether minimal alcohol consumption can have beneficial effects on blood pressure [20]. Roerecke and colleagues observed that any daily amount of alcohol consumed, even minimal amounts, had no protective effects on blood pressure [21]. Similar results were achieved in a systematic review with a meta-analysis conducted by Jung and colleagues; they concluded by stating that any amount of alcohol consumed daily is linked to an increased risk of developing systemic arterial hypertension [22]. Bulpitt considered the question of whether elderly people with hypertension should continue to drink alcohol; in his work, he highlighted that subjects over 60 years of age who consumed more than 16 drinks a week should reduce their alcohol consumption but not abolish it definitively, underlining that daily consumption could be beneficial [23]. A significant reduction in alcohol consumption is essential to improve blood pressure levels, associated with correct pharmacological therapy, with ACE inhibitors, diuretics, and agents that block calcium channels; significant improvements in blood pressure can be observed after just over one month of abstinence from alcohol, with a reduction of 7.2 mmHg in mean arterial pressure in subjects who consume large quantities of alcohol. The same decrease in blood pressure was not observed in subjects who consumed two drinks or less per day; the American Society of Hypertension and the International Society of Hypertension recommend that men drink no more than two drinks per day and one per day for women [11,12,24,53].

### 4.2. Alcohol Consumption and Cardiac Arrhythmias

Consuming alcohol in moderate amounts has been linked to a lower risk of cardiovascular death, possibly due to alcohol’s anti-ischemic effects, in contrast to those who abstain entirely. However, the consumption of alcohol in large quantities relates to a variety of cardiac irregularities that can lead to the development of cardiac arrhythmias. These include changes in heart structure, disturbances in electrolyte levels, an extended QT interval on the electrocardiogram, increased activity of the sympathetic nervous system, a decrease in the variability of heart rate, and diminished sensitivity of baroreceptors. These alterations, induced by excessive alcohol intake, can significantly impact the electrical activity of the heart [6,25,54].

Researchers have studied the proarrhythmic effects of alcohol in various animal models; a study conducted on a canine model showed that alcohol consumption reduced the heart rate, prolonged the H–Q interval and the QRS complex, and delayed the conduction of the electrical impulse from the bundle of His to the left branch [26].

Another research group investigated cardiac alterations on an animal model resulting from alcohol consumption, highlighting, in a population of rats that consumed 39% of their calories as ethanol for approximately 12 months, both altered myocardial contractility and a defective relaxation. The same study also analyzed a population of dogs in which 39% of the calories consumed were alcohol, observing a rapid reduction in left ventricular compliance. In both animal populations studied, an early increase in myocardial collagen was observed, mainly localized in the perivascular and intermyofibrillar areas, associated with hepatic alterations; this evidence suggested to researchers that the accumulation of extracellular collagen may be responsible for the reduction in cardiac diastolic compliance [55]. A surprising result was obtained from a study conducted on some monkeys in which 40% of the calories consumed were represented by alcohol for a period of four months, in which the researchers histologically highlighted myocytolysis and myocardial fibrosis [56].

In humans, both the consumption of large quantities of alcohol sporadically, in the so-called “alcoholic binge”, and the chronic consumption of alcohol predispose to the onset of cardiac arrhythmias. The consumption of large quantities of alcohol in so-called binges is associated with the development of cardiac arrhythmias, even in subjects with normal and functioning myocardial tissue. The term “holiday heart” was coined by Ettinger in 1978 when he described abnormalities in the heart rhythm or impulse conduction system associated with the consumption of large quantities of alcohol in subjects who had no clinical evidence of heart disease. These ECG alterations ceased with alcoholic abstinence. Furthermore, these arrhythmias associated with alcohol consumption had a seasonal prevalence, with the period of maximum incidence during the Christmas holidays (Christmas and New Year). This syndrome had been described as an “acute arrhythmic event caused by excessive alcohol consumption in subjects who had no clinical or structural evidence of cardiac anomalies, which disappears with alcohol abstinence” [3,12,18,27,50,57].

It is estimated that this syndrome is responsible for 35–62% of cases of atrial fibrillation induced by alcohol abuse. At a molecular level, alcoholic binges appear to exert a proarrhythmic effect through the activation of the atrial c-Jun-N-terminal kinase (JNK) with consequent stimulation of the phosphorylation of Calmodulin Kinase II (CaMKII), increased cytoplasmic calcium and increased susceptibility to atrial arrhythmias [12,27].

The most frequent cardiac arrhythmia in this class of patients is atrial fibrillation, although physicians recorded episodes of atrial flutter, junctional tachycardia, isolated premature ventricular/atrial complexes, paroxysmal atrial tachycardia, and ventricular tachycardia. Furthermore, it is important to underline how alcohol can act as a co-factor associated with a pre-existing electrolyte alteration or acute pathologies (infections), determining the generation of supraventricular arrhythmias [28,58].

The risk of developing ventricular tachycardia is higher in subjects who consume large quantities of alcohol (21–35 drinks per week) or in binge episodes compared to subjects who consume moderate quantities (2–6 drinks per week) [29,59,60].

Chronic alcohol consumption is a predisposing factor for the onset of cardiac arrhythmias through a dual mechanism represented by electrolyte and vitamin alterations (such as hypomagnesemia, hypophosphatemia, and beriberi) and by alcoholic cardiomyopathy.

Hypomagnesemia is characteristically associated with chronic alcoholism; a combination of renal and gastrointestinal alterations represents the mechanisms underlying this ionic deficiency. Alterations to normal cardiac rhythm are frequently highlighted in patients with hypomagnesemia, and the administration of magnesium improves cardiac mechanical recovery in patients with myocardial infarction. Furthermore, it was observed that the administration of magnesium in subjects with potentially lethal cardiac arrhythmias corrected these arrhythmias. In addition to magnesium, hypophosphatemia is another ion deficiency observed in subjects with chronic alcoholism; alcohol induces hypophosphatemia through at least three mechanisms: increased renal loss of phosphate groups, the metabolic acidosis that characterizes these patients, which further aggravates the renal losses, and finally gastrointestinal losses caused by malabsorption, diarrhea, vomiting and abuse of antacids. Studies on animal models have highlighted an alteration to normal myocardial function in animals with phosphate deficiency [27,51,59,61].

The insult to myocardial tissue caused by alcohol triggers cardiac reparative mechanisms that try to minimize cardiac damage. Still, being limited, they are ineffective in chronic alcoholic subjects [61]. Histological alterations to cardiac tissue are characterized by myocardial necrosis, replacement fibrosis, and hypertrophy of surviving myofibrils; in this scenario, subjects with a left ventricular ejection fraction less than 15% develop frequent episodes of congestive heart failure and ventricular arrhythmias. Furthermore, the continuous cardiac fibrotic process, essential in the repair processes of damaged tissues, causes myocardial fibrosis with a consequent increase in cardiac parietal stiffness, reduction in myocardial tissue oxygenation, and increased risk of arrhythmias [12,30,31,32,50,62,63,64].

According to a Korean study conducted on 9,776,956 patients, weekly frequency of alcohol consumption and quantity is statistically correlated with the development of atrial fibrillation [12,65].

The cardiac anatomical alterations in subjects with alcoholic cardiomyopathy are dilation of the left ventricle, dilation of both ventricles and reduction in ventricular wall thickness. The prevalent arrhythmias in this class of subjects are atrial fibrillation and flutter, although atrioventricular blocks, premature ventricular contractions and left bundle branch block may occur. Sulaiman and colleagues analyzed, in a cohort of subjects suffering from alcoholic cardiomyopathy, the independent factors predictive of the onset of atrial fibrillation, highlighting age, obstructive sleep apnea, hypothyroidism, Caucasian race, and obesity; the same group of researchers concluded their analysis represented that cardiac arrhythmias were the cause of hospitalization in approximately 48% of patients with alcoholic cardiomyopathy [30,31,32,33,50,66]. The changes observed on electrocardiograms in dogs with chronic alcoholism have been correlated with a high incidence of sudden death [26,33,62].

Another potentially fatal arrhythmia observed in subjects with alcoholic cardiomyopathy is prolongation of the QT interval. This prolongation of the QT interval can further worsen due to the intake of poly-substances, such as psychotropic drugs (tricyclic antidepressants, selective serotonin reuptake inhibitors, lithium, and methadone), a frequent occurrence in subjects with alcohol dependence [67,68,69,70].

A study conducted by Guzzo-Merello found that left bundle branch block was an independent prognostic factor for malignant ventricular arrhythmias in patients with alcoholic cardiomyopathy. Furthermore, the onset of ventricular arrhythmias was not observed in patients with a left ventricular ejection fraction > 40% or NYHA class 1 [69].

A large study conducted on a population of 109,230 participants in northern Sweden, of which 5230 were experiencing an episode of atrial fibrillation, highlighted in men that the consumption of increasing quantities of alcohol was associated with an increased risk of developing this cardiac arrhythmia; the same association was not highlighted in women [34]. Another study conducted on 47,002 participants in Norway showed a hazard ratio in subjects who consumed >7 drinks per week of 1.38 compared to subjects who did not consume alcohol [27]. A study conducted on 47,002 participants in Norway showed a hazard ratio in subjects who consumed >7 drinks per week of 1.38 compared to subjects who did not consume alcohol [35].

### 4.3. Alcohol Consumption and Sudden Death

**Figure 2:**
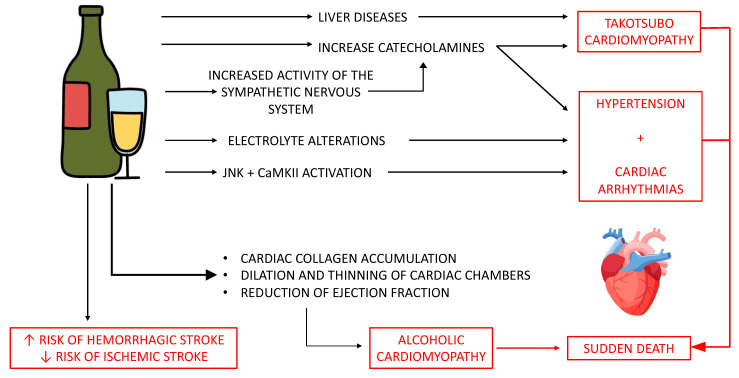
Significant findings on alcohol consumption and sudden death. Pathophysiology of alcohol intake leading to sudden death.

As illustrated above, anatomical and structural alterations observed in patients suffering from alcoholic cardiomyopathy predispose to the onset of ventricular arrhythmias and sudden cardiac death [71,72] (Figure 2). Between 30 and 40 percent of people with alcoholic cardiomyopathy develop sudden cardiac mortality [73].

While acute alcohol consumption is considered a predisposing factor for potentially fatal cardiac arrhythmias such as Brugada syndrome, chronic alcohol consumption, when it comes to the habitual consumption of large quantities of alcohol, could cause supraventricular arrhythmias, most commonly atrial fibrillation, and potentially fatal ventricular arrhythmias [27,30,69].

Experimental work conducted on mice observed that hemodynamically significant changes in alcoholic cardiomyopathy were observed after 20 weeks of regular alcohol consumption, with a reduction in the ejection fraction of the left ventricle. Furthermore, this reduction in cardiac inotropic function was accompanied by dilation of the cardiac chambers. The decrease in cardiac ejection fraction in these rats predisposed to the occurrence of sudden cardiac death [74].

A group of researchers analyzed autopsies of subjects who died from sudden cardiac death and found that, excluding subjects with severe coronary artery disease, there were subjects who did not have coronary atherosclerosis sufficient to justify sudden cardiac death but who had a positive history of prolonged and systematic intake of alcohol and steatohepatitis; the researchers emphasized that, among those who died from alcoholic cardiomyopathy, only 8.4% had superior coronary stenosis, with a reduction in the vascular lumen by at least half (stenosis of at least 50% of the lumen) [36,37,75].

The Fingesture study found that, out of 5869 autopsies performed on subjects who died from sudden cardiac death, 290 (4.9% with an average age of 56 years, males in 83% of cases) were killed due to alcoholic cardiomyopathy. Of these 290, 77.9% of patients had never been diagnosed with a heart disease in life. Of these, 94.5% of cases (274 out of 290) presented hepatic steatosis, and 64.5% (187/290) hepatic cirrhosis. In subjects who died from sudden cardiac death related to dilated cardiomyopathy and had been diagnosed with cardiac pathology (64 out of 290, 22.1%), the most present risk factors were represented by hypertension, type 2 diabetes mellitus, and hypercholesterolemia. Alcohol-related diseases, such as alcoholic liver disease, liver cirrhosis, pancreatitis, and convulsions, were only minimally diagnosed in subjects who died from sudden cardiac death secondary to dilated cardiomyopathy. This study concluded by stating that the majority of patients who died from sudden cardiac death secondary to alcoholic cardiomyopathy had never received a diagnosis of heart disease; on the contrary, they had been diagnosed as habitual consumers of large quantities of alcohol or as alcoholics [36].

Although alcoholic cardiomyopathy represents only 3.8% of all cardiomyopathies, it is responsible for 16–19% of sudden cardiac deaths; furthermore, alcoholic cardiomyopathy is the most common cause of sudden cardiac death from non-ischemic causes between the ages of 40 and 59 (25.8%) [37,38,39,76,77,78]. Vikhert and colleagues revealed that, out of 7052 autopsies of subjects who died from sudden cardiac death, 17% of the subjects suffered from dilated cardiomyopathy and had a history of alcohol abuse, and the majority were male and under 50 years of age (73%) [38]. This researcher suggested that the prevalence of alcoholic cardiomyopathy as a cause of death may be underestimated; furthermore, it is essential to consider how both alcohol itself and abstinence from alcohol consumption are considered factors favoring the onset of arrhythmias such as ventricular fibrillation and atrial fibrillation.

In patients with alcoholic cardiomyopathy, negative prognostic factors for cardiac events include the absence of treatment with beta-blockers or digoxin, a history of atrial fibrillation, QRS width greater than 120 ms, and a short distance in a walking test lasting 6 min. Furthermore, it was observed that neither previous average consumption of alcohol, nor the duration of abuse, nor the type of alcoholic beverage consumed were prognostic indicators [40]. Furthermore, it was highlighted that 30–40% of subjects with alcoholic cardiomyopathy died from sudden cardiac death [38,39,74].

Hookana and colleagues analyzed the causes of death of 579 subjects who died from sudden cardiac death; they highlighted that in 19% of cases the cause of death was due to alcoholic cardiomyopathy, which represents the second cause of sudden cardiac death after only obesity (23.7%) [41]. Table 2.

### 4.4. Alcohol Consumption and Stroke

The scientific community has demonstrated how the relationship between alcohol and stroke is complicated by numerous etiological factors predisposing the onset of stroke; drinkers of large quantities of alcohol have an (apparently) higher risk of hemorrhagic stroke, unlike moderate drinkers who show no risk or, on the contrary, show a protective role against ischemic stroke (Figure 1) [41,49,78]. A meta-analysis conducted on 23 studies (29,457 participants) showed that moderate alcohol consumption has a protective role against myocardial infarction and hemorrhagic stroke, with a 30% reduction in the risk of ischemic stroke within a week; by contrast, high alcohol consumption is associated with high immediate and within-a-week cardiovascular risk [79].

The intake of high quantities of alcohol causes cirrhosis, convulsions, stroke, and malignant pathologies such as cancer of the colon and rectum, breast, larynx, and liver; instead, the habitual consumption of moderate quantities of alcohol is linked to a lower risk of developing diabetes mellitus, stroke, heart failure and any cause of death. Several research groups have explored the pathogenic role of alcohol in the development of stroke. Overall, these studies highlighted that the consumption of 1–2 drinks per day determines a reduction in some types of stroke (J-shaped association) [11,18,42,80,81].

Alcohol consumption in moderate quantities has a protective effect on the development of ischemic stroke, heart failure, increase in HDL, and reduction in LDL with a related decrease in atherosclerotic risk [11,40]. A study conducted on 47,000 Japanese women showed that alcohol consumption greater than or equal to 300 g per week is associated with a twofold higher risk of stroke. The American Stroke Association guidelines recommend that high-drinking patients with ischemic stroke or TIA should eliminate or reduce their drinking and define alcohol consumption as “reasonable” at two drinks per day for men and one for women [11].

In both Scandinavia and Europe, an increased risk of hemorrhagic stroke and ruptured subarachnoid aneurysms has been observed in subjects who consume high quantities of alcohol. Furthermore, alcoholic binges are associated with the onset of stroke-like episodes within 24 h; some authors have suggested that excessive alcohol consumption may predispose to the risk of stroke and sudden death [19,42,43,44,80,81,82].

One study suggests that the antithrombotic effect of alcohol would protect against the risk of ischemic stroke while increasing the risk of hemorrhagic stroke; the same authors highlighted a greater risk of hemorrhagic stroke in subjects who consume large quantities of alcohol and not in those who drink small or medium quantities, probably caused by an increase in systemic blood pressure [19,42].

### 4.5. Alcohol Consumption and Takotsubo Cardiomyopathy

Takotsubo cardiomyopathy (TC, also known as heart apex swelling syndrome, broken heart syndrome, and stress-induced cardiomyopathy) is a clinical condition characterized by a sudden onset of left ventricular dysfunction that occurs following intense stress with elevation in circulating catecholamines, such as emotional stress, surgery, intracranial bleeding, ischemic stroke, drug administration, sepsis, liver disease (liver cirrhosis) and hepatic encephalopathy [45,83].

Although physicians describe a benign and transient clinical course in 90% of cases, this syndrome is often associated with electrical instability, cardiogenic shock, ventricular arrhythmias, heart failure, and pulmonary edema, characterized by high morbidity and mortality. Several etiological theories have been proposed to explain the pathogenesis of Takotsubo syndrome; the excess of circulating catecholamines, microvascular dysfunction, and intense coronary spasms have been taken into consideration, but the exact pathogenetic mechanism has not yet been understood [46,47,84,85,86,87,88]. Excess catecholamines directly affect cardiomyocytes with myocardial stunning (stunning), coronary vasospasm, myocardial ischemia, microcirculatory dysfunction, and left ventricular dysfunction [46,85,88].

Clinically, TC manifests itself early, simulating myocardial infarction with chest pain, dyspnea, and syncope. Still, the absence of coronary pathology on angiographic examination helps to differentiate the two pathological entities [46,84,86,88,89].

The diagnostic criteria established for TC by the Mayo Clinic in 2008 [84,85] are:

- Transient left ventricular systolic dysfunction (akinesia, dyskinesia or hypokinesia);
- Absence of coronary artery disease or rupture of atherosclerotic plaque. If coronary artery disease is identified, the wall anomalies observed must not be tributary areas of the coronary artery involved;
- New onset ECG abnormalities (ST elevation, T wave inversion, new shoulder blocks) or modest increase in cardiac troponin;
- Absence of pheochromocytoma (which would explain the high level of circulating catecholamines) or myocarditis.

In the scientific literature, there is no reference to a possible etiological role of alcohol consumption in Takotsubo cardiomyopathy. In the studies included in this review, as previously mentioned, liver disease and elevated circulating catecholamine levels were considered two probable etiological factors in TC.

We know that both liver disease and elevated catecholamines are related to alcohol abuse; furthermore, in the studies analyzed, there are case reports of the onset of Takostubo cardiomyopathy in subjects with liver transplantation [46,85,86,87], acute pancreatitis [47,48], liver cirrhosis [48,89], and cardiogenic shock [90]. A pathogenetic role of alcohol in Takostubo syndrome is possible, considering that in the cases described above, the pathologies connected to the onset of Takostubo cardiomyopathy are, in turn, connected to a positive history of alcohol abuse.

Finally, we can exclude a relationship between alcoholic cardiomyopathy and Takotsubo cardiomyopathy; in the case of Takotsubo pathology, the cardiac anatomical alterations are, by definition, transitory, while by contrast the structural cardiac alterations during alcoholic cardiomyopathy are irreversible.

### 4.6. Demographic Matters in Alcoholic Cardiomiopathy

Alcoholic cardiomyopathy exhibits distinct demographic patterns that have been observed across various studies. This condition predominantly affects middle-aged individuals, with a slight male predominance, reflecting broader alcohol consumption patterns. Epidemiological data suggest that the risk of developing alcoholic cardiomyopathy increases with the duration and quantity of alcohol intake, making heavy, long-term drinkers particularly susceptible. Socioeconomic factors also play a critical role, as lower socioeconomic status is often associated with higher levels of alcohol abuse and, consequently, a greater risk of alcoholic cardiomyopathy. Additionally, genetic predispositions may influence the susceptibility of certain populations to this condition. Geographic variations in the prevalence of alcoholic cardiomyopathy have been noted, potentially due to differences in drinking cultures and alcohol availability. Understanding these demographic aspects is crucial for targeting preventive measures and tailoring treatment approaches for those most at risk [41,91].

The specific demographic trends of alcoholic cardiomyopathy can be summarized based on available research and epidemiological data:

Alcoholic cardiomyopathy commonly affects individuals in middle age, often between the ages of 35 and 65. This range correlates with the duration of alcohol abuse required to develop the condition. There is a higher prevalence among males compared to females. This difference is partly attributed to higher rates of heavy alcohol consumption among men, although recent data suggest that the gender gap in alcohol use and its health effects is narrowing in some populations. Lower socioeconomic status is associated with higher rates of chronic alcohol consumption and, consequently, a greater risk of developing alcoholic cardiomyopathy. Factors such as stress, lack of access to healthcare, and economic instability may contribute to increased alcohol use in these populations [92]. The incidence of alcoholic cardiomyopathy varies globally, influenced by cultural attitudes towards alcohol, availability, and consumption patterns. Countries with high per capita alcohol consumption tend to report higher rates of the condition. Some studies suggest ethnic and racial differences in susceptibility to alcoholic cardiomyopathy, which could be due to genetic factors, differences in alcohol metabolism, and social determinants of health [93].

It is important to note that while these trends provide a general overview, individual risk can be influenced by a variety of factors including genetic predisposition, personal health history, and lifestyle choices. Public health efforts to reduce the incidence of alcoholic cardiomyopathy focus on education about the risks of heavy drinking, promoting alcohol moderation or abstinence, and providing support for individuals with alcohol use disorders [94].

## 5. Conclusions

This comprehensive review highlights the pathophysiological alterations related to alcohol consumption in different ways, both in chronic alcohol consumers and in the case of alcohol binges [95]. In this regard, population and gender studies can add further significant knowledge [96,97,98,99] to provide fundamental support not only in clinical medicine (to undertake pharmacological therapies for alcohol-consuming patients with unknown cardiac alterations), but also in forensic medicine diagnosis of causes of death. Especially in the case of cardiac sudden death, it is crucial to discriminate whether the histological findings can be attributable to dilated cardiomyopathy or alcoholic cardiomyopathy, which often have histologically similar characteristics.

In addition, these study outcomes could be an essential tool to highlight the correlation between alcohol and arrhythmias, which can be fatal. Further prospective studies are necessary to improve knowledge of risk factors for the development of structural and molecular modifications of vascular endothelium and cardiac structure [100,101,102].

In conclusion, alcoholic cardiomyopathy represents a significant and severe health concern directly linked to chronic and excessive alcohol consumption. This condition underscores the critical impact of lifestyle choices on cardiovascular health, manifesting in weakened heart muscles, diminished cardiac function, and a host of life-threatening complications such as heart failure and arrhythmias.

## 6. Future Directions

The demographic trends associated with alcoholic cardiomyopathy, including its prevalence among middle-aged individuals and a notable male predominance, highlight the importance of targeted public health interventions and awareness campaigns. Furthermore, the socioeconomic factors and geographical variations in its occurrence call for a multifaceted approach in addressing alcohol abuse and its health implications. Alcoholic cardiomyopathy not only exemplifies the dire consequences of sustained alcohol abuse on the heart but also emphasizes the broader societal and healthcare challenges posed by alcohol-related disorders. As research continues to elucidate the pathophysiological mechanisms and risk factors of this condition, it is imperative that healthcare systems and communities work together to mitigate the impact of alcohol abuse through education, support services, and policy measures aimed at reducing alcohol consumption and promoting cardiovascular health.

## Figures and Tables

**Table 1:** Notable features of 39 relevant studies summarized.

Mesh Terms	Authors	Study Design	N° Subjects	Outcomes
Alcohol consumption and blood pressure	O’Keefe, 2014 [11]	Review	///	Drinking more than two drinks in a day raises the risk of hypertension.
	Stătescu, 2021 [12]	Review	///	Any daily amount of alcohol consumed, even minimal amounts, had no protective effects on blood pressure.
	Knochel, 1983 [13]	Review	///	In chronic alcoholics without clinical evidence of heart disease, a blood ethanol level of 150 mg/dL elevated left ventricular end diastolic pressure and decreased stroke volume.
	Lee, 2002 [14]	Review	///	Consumption of more than 40 g of ethanol per day increases blood pressure. Excessive alcohol consumption is responsible for 7% of all hypertension cases.
	Piano, 2020 [15]	Review	///	In women the consumption of 1–2 drinks a day does not affect blood pressure levels.
	Roerecke, 2018 [16]	Systematic review and meta-analysis	326,254	Any alcohol consumed in men increases the risk of hypertension. In women, there was no risk of hypertension for consumption of 1 to 2 drinks/day.
	Davidson, 1989 [17]	Observational study	83,947	Male subjects who consumed <2 drinks per day had no increase in blood pressure levels compared with controls, while women who consumed <2 drinks per day had lower blood pressure values than controls.
	Klatsky, 2015 [18]	Review	///	The hypertensive effect caused by alcohol disappears four days after the last intake.
	Clark, 1984 [19]	Review	///	Women who drink alcohol in moderation have mean blood pressures that are lower than those of teetotalers.
	Husain, 2014 [20]	Review	///	After a month of alcohol abstinence, a 7.2 mmHg drop in mean arterial pressure occurred in heavy drinkers.
	Roerecke, 2017 [21]	Systematic review and meta-analysis	2865	In individuals who consumed more than two drinks daily, reducing alcohol consumption was linked to a higher reduction in blood pressure.
	Jung, 2020 [22]	Systematic review and meta-analysis	86,188	Any alcohol consumption, even in quantities less than 20 g per day, is associated with an increased risk of hypertension.
	Bulpit, 2005 [23]	Review	///	Systolic pressure decreased by 3.3 mmHg and diastolic pressure decreased by 2.0 mmHg with a 76% reduction in alcohol consumption.
	Kloner, 2007 [24]	Review	///	There was a higher incidence of hypertension in individuals who drank three or more drinks per day.
Alcohol consumption and cardiac arrhythmias	Manolis, 2022 [25]	Retrospective observational study	47,002	HR for AF of 1.38 when comparing participants consuming >7 drinks per week with abstainers.
	Ettinger, 1976 [26]	Animals experimental study	///	In guinea pigs, prolongation of the H–Q interval and the QRS complex reflects the duration of alcohol exposure and the quantity consumed.
	Giannopoulos, 2022 [27]	Meta-analysis	///	Atrial fibrillation is the most frequent cardiac arrhythmia both in chronic alcohol consumers and in alcoholic binges.
	Greenspon, 1983 [28]	Human experimental study	14	In 71% of cases, consumption of 90 mL of 80-proof whiskey resulted in sustained or nonsustained atrial or ventricular tachyarrhythmias.
	Fauchier, 2003 [29]	Observational study	75	In patients with alcoholic cardiomyopathy, alcohol abstinence significantly reduces cardiac arrhythmic events.
	Fernàndez-Solà, 2016 [30]	Review	///	The consumption of more than five drinks induces an acute decrease in myocyte contractility and arrhythmia and may cause sudden death.
	Sulaiman, 2020 [31]	Observational study	75,430	In patients with alcoholic cardiomyopathy, cardiac arrhythmias occur in 48% of cases, and 10% of cases are the cause of hospitalization.
	Bashour, 1975 [32]	Observational study	65	Premature ventricular contractions and first-degree atrioventricular blocks are the main ECG alterations in patients with alcoholic cardiomyopathy, occurring in 37% and 34% of cases, respectively.
	Kim, 2020 [33]	Observational study	9,776,956	The amount of alcohol taken during each drinking session was not an independent risk factor for new-onset atrial fibrillation. Still, frequent drinking and weekly alcohol intake were significant risk factors.
	Guzzo-Merello, 2015 [34]	Retrospective observational study	94	Ventricular arrhythmias did not occur in patients with alcoholic cardiomyopathy and ejection fraction > 40% or NYHA class 1.
	Johansson, 2020 [35]	Observational study	109,230	The consumption of increasing quantities of alcohol was associated with an increased risk of developing this cardiac arrhythmia.
Alcohol consumption and sudden death	Hietanen, 2019 [36]	Retrospective observational study	5869	Of patients who died from alcoholic cardiomyopathy, 77.9% had never received a diagnosis of cardiac pathology.
	Haukilahti, 2019 [37]	Retrospective observational study	5869	Alcoholic cardiomyopathy is responsible for 5.3% and 4% of sudden cardiac deaths in men and women, respectively.
	Vikhert, 1986 [38]	Observational study	752	Among subjects who died from sudden cardiac death, 17% had alcohol abuse, and the majority were under 50 years of age.
	Guzzo-Merello, 2015 [39]	Observational study	282	In this 59-month observational study, 8.5% of patients with alcoholic cardiomyopathy died due to sudden cardiac death.
	Laurent, 2022 [40]	Review	///	Alcoholic cardiomyopathy is responsible for 16–19% of sudden cardiac deaths and is the leading cause of non-ischemic cardiac death in subjects aged 40–59 years.
	Hookana, 2011 [41]	Observational study	2661	A total of 19% of deaths from sudden cardiac death were caused by alcoholic cardiomyopathy, representing the second cause of death after obesity (23.7%).
Alcohol consumption and stroke	O’Keefe, 2014 [11]	Observational study	47,100	In women, consuming more than or equal to 300 g of alcohol each week is linked to a two-fold increased risk of stroke.
	Mostofsky, 2016 [42]	Systematic review and meta-analysis	29,457	The consumption of six drinks per week reduces the risk of ischemic stroke by 19%; however, there is a 2.25-fold risk of onset of ischemic stroke with the consumption of 19 drinks per week.
	Milic, 2016 [43]	Review	///	Excessive alcohol consumption is associated with the onset of stroke-like episodes within 24 h, suggesting that excessive alcohol consumption may predispose to the risk of stroke and sudden death.
	Klatsky, 2010 [44]	Review	///	There is a greater risk of hemorrhagic stroke in subjects who consume large quantities of alcohol than in those who drink small or medium quantities, probably caused by an increase in systemic blood pressure.
Alcohol consumption and Takostubo cardiomyopthy	Angelini, 2021 [45]	///	///	In the scientific literature, there is no reference to a possible etiological role of alcohol consumption in Takotsubo cardiomyopathy. Liver disease and elevated circulating catecholamine levels were considered two probable etiological factors in Takotsubo cardiomyopathy.
	Luu, 2020 [46]			
	Yeh, 2021 [47]			
	Al Juboori, 2016 [48]			

**Table 2:** Significant findings on alcohol consumption and cardiac sudden death (SCD).

Author	Epidemiology Data	Pathology Findings	Cardiac Sudden Death
Hietanen S, et al. [36]	subset of Fingesture cohort; period: 1998–2017, Northern Finland, autopsy study	Alcoholic cardiomyopathy	4.9% of patient cohort
Hietanen S, et al. [36]	5869 autopsies performed on subjects who died from sudden cardiac death	77.9% of patients had never been diagnosed with a heart disease in life. The most present risk factors: hypertension, type 2 diabetes mellitus, and hypercholesterolemia.	
Corović N, et al. [73]	Clinical study of changes in the cardiovascular system of alcoholic patients admitted for control of their health status and evaluation of their working ability	Alcohol abuse is related to significantly higher dispersions of the QTc and JTc intervals and thus a significantly higher estimation of relative risk for prolonged QTc interval and higher QTc dispersion than the control group, i.e., higher risk of arrhythmias.	Alcohol-related cardiac arrhythmias are more often the cause of death, due to the prolongation of the QT interval, than are the consequences of coronary atherosclerosis.
Hookana E, et.al [41]	2661 consecutive victims of SCD (Northern Finland) included in the study: autopsies plus available medical records and standardized questionnaires.	CM related to obesity, fibrotic CM, and alcoholic CM are commonly associated with nonischemic SCD.	Alcoholic CM accounted for (19.0%) of all SCD cases; alcoholic CM was the most common cause of death in subjects between 40 and 59 years of age (25.8%)
Roshchevskaya IM, et al. [74]	Animal model	Reduction in cardiac inotropic function was accompanied by dilation of the cardiac chambers	Electric instability, malignant heart rhythm disturbances

## Data Availability

All datasets analyzed and generated during this research will be shared upon request.
